# Dsg1 and Dsg3 Composition of Desmosomes Across Human Epidermis and Alterations in Pemphigus Vulgaris Patient Skin

**DOI:** 10.3389/fimmu.2022.884241

**Published:** 2022-05-25

**Authors:** Thomas Schmitt, Julia Pircher, Letyfee Steinert, Katharina Meier, Kamran Ghoreschi, Franziska Vielmuth, Daniela Kugelmann, Jens Waschke

**Affiliations:** ^1^Chair of Vegetative Anatomy, Instiute of Anatomy, Faculty of Medicine, Ludwig-Maximilian-Universität München (LMU) Munich, München, Germany; ^2^Department of Dermatology, Venereology and Allergology, Charité-Universitätsmedizin Berli, Corporate Member of Freie Universität Berlin, Humboldt-Universität zu Berlin, and Berlin Institute of Health, Berlin, Germany

**Keywords:** pemphigus, dermatology, desmoglein, desmosome, adhesion, epidermis, skin, autoimmune disease (AD)

## Abstract

Desmosomes are important epidermal adhesion units and signalling hubs, which play an important role in pemphigus pathogenesis. Different expression patterns of the pemphigus autoantigens desmoglein (Dsg)1 and Dsg3 across different epidermal layers have been demonstrated. However, little is known about changes in desmosome composition in different epidermal layers or in patient skin. The aim of this study was thus to characterize desmosome composition in healthy and pemphigus skin using super-resolution microscopy. An increasing Dsg1/Dsg3 ratio from lower basal (BL) to uppermost granular layer (GL) was observed. Within BL desmosomes, Dsg1 and Dsg3 were more homogeneously distributed whereas superficial desmosomes mostly comprised one of the two molecules or domains containing either one but not both. Extradesmosomal, desmoplakin (Dp)-independent, co-localization of Dsg3 with plakoglobin (Pg) was found mostly in BL and extradesmosomal Dsg1 co-localization with Pg in all layers. In contrast, in the spinous layer (SL) most Dsg1 and Dsg3 staining was confined to desmosomes, as revealed by the co-localization with Dp. In pemphigus patient skin, Dsg1 and Dsg3 immunostaining was altered especially along blister edges. The number of desmosomes in patient skin was reduced significantly in basal and spinous layer keratinocytes with only few split desmosomes found. In addition, Dsg1-Pg co-localization at the apical BL and Dsg3-Pg co-localization in SL were significantly reduced in patients, suggesting that that extradesmosomal Dsg molecules were affected. These results support the hypothesis that pemphigus is a desmosome assembly disease and may help to explain histopathologic differences between pemphigus phenotypes.

## Introduction

**Figure 1:**
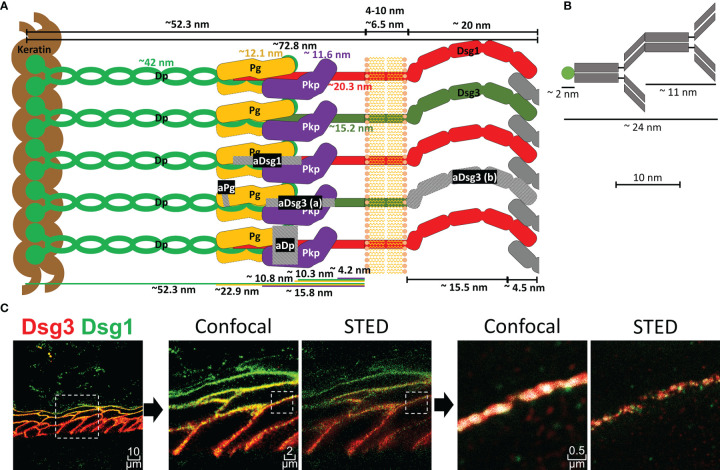
**(A)** Schematic depiction of a desmosome unit sub-cell with all proteins shown to scale as indicated. Grey hatched areas mark regions against which antibodies used in this study were raised. aDsg3 (a) is a rabbit IgG and aDsg3 (b) a mouse IgG, as indicated in the materials and methods section. **(B)** Depiction of a primary and dye coupled secondary antibody complex for scale. **(C)** on the right is an overview of a Dsg1 (green)-Dsg3 (red) staining with 3x zoom and 15x zoom to a single cell border comparing the co-localization results using confocal images vs. super resolution STED images (squares with dotted borders mark the zoomed in region).

Desmosomes are one of the most important cell-cell contacts for the mechanical integrity of the epidermis (1–3). The general structure of desmosomes is well known. They consist of transmembrane adhesion proteins from the cadherin family which adhere to partner molecules from neighbouring cells in both homo- and heterophilic manner (4). The desmosomal cadherins are connected to the intracellular plaque, which is linked to the keratin cytoskeleton (5, 6) (**Figure 1A**), and comprise desmogleins (Dsg)1-4 and desmocollins (Dsc)1-3 (1). The plaque proteins include plakoglobin (Pg), plakophilin (Pkp)1-3 and desmoplakin (Dp). Dsg1 and Dsc1 are more prominent in superficial epidermal layers, while Dsg3 and Dsc3 are more dominantly expressed in lower layers. Dsg4 on the other hand is reported to be missing in basal cells and only found in the granular layer and hair follicles (7). Starting from the granular layer, corneodesmosin starts additionally linking cadherins together in the extracellular space (7). This shift in composition marks the stepwise maturation of adaptable desmosomes towards stable corneodesmosomes found in the cornified layer. In mucosal epithelium, the situation is slightly different. Dsg2 is found in the basal layers only (8). Dsg1 is less predominant and particularly absent in the basal layer of mucosa whereas Dsg3 is present in all layers (8). In contrast, Dsc1 is not present in oral mucosa while Dsc2 and Dsc3 are present in all layers (1).

Pemphigus is a severe autoimmune blistering skin disease disrupting desmosomes and is caused by autoantibodies predominantly against Dsg1 and Dsg3, which finally compromises the integrity of epidermal and mucosal tissue (9, 10). However, beside the reports of altered contents of desmosomal components along different epidermal layers (7) and changes in the distribution of cytoskeleton-bound and not cytoskeleton-bound fractions caused by pemphigus autoantibodies (11–13), little is known about detailed changes in the composition of desmosomes regarding the pemphigus autoantigens Dsg1 and Dsg3 during maturation of keratinocytes across the epidermal layers. The main three pemphigus variants are: Muco-cutaneous pemphigus vulgaris (PV) which shows antibodies against Dsg1 and Dsg3 and affects the epidermis and the mucosa. About half of the PV patients suffer from mucosal-dominant PV, which is characterized by antibodies against Dsg3 and restricted to the mucosa only. Finally, pemphigus foliaceus (PF) with antibodies against Dsg1 causes acantholysis in the epidermis only (1, 14). Strikingly, in PV epidermal cleavage occurs at the basal-suprabasal interface whereas in PF splitting occurs predominantly within the granular layer (15). These different histopathologic findings can in part be explained by the difference in distribution of cadherins according to the desmoglein compensation theory (16). However, Dsg compensation alone is not sufficient to explain why in PV autoantibodies against Dsg1 and Dsg3 cause acantholysis at the suprabasal interface but not between other epidermal layers. Intracellular signalling was shown to be important for pemphigus pathogenesis (17). Since it was shown that signalling pathways are at least in part specific for Dsg1 and Dsg3 (17–19), this may affect the clinical phenotype, especially when the distribution of Dsg1 and Dsg3 inside and outside of desmosomes changes for different epidermal layers.

This study characterizes the molecular composition of desmosomes and extradesmosomal contacts in different epidermal layers with a focus on the pemphigus autoantigens Dsg1 and Dsg3 as well as changes observed in PV patient skin using immunostaining in combination with super resolution stimulated emission depletion (STED) microscopy.

## Results

### Super Resolution Microscopy Shows the Composition of Desmosomes Within the Human Epidermis With High Precision

To perform co-localization analysis using super-resolution STED (stimulated emission depletion) microscopy, antibodies against several proteins of interest including Dsg1, Dsg3, Pg and Dp were selected. **Figure 1** depicts a representation of a desmosomal sub-unit, showing the localization of each protein as described in the literature, sizes and distances depicted are mostly based on the work of North et al. (20). To sensitively detect co-localization of the proteins, antibodies against epitopes in close proximity within the desmosomal intracellular region were selected (**Figure 1A**). Only exception was a mouse anti-Dsg3-IgG (aDsg3(b), **Figure 1A**), which was used for co-staining with the rabbit anti-Dsg1-IgG. It was the only suitable mouse anti-Dsg3-IgG available to us and was directed against the extracellular region of Dsg3 instead. For all other experiments, the rabbit anti-Dsg3 IgG (aDsg3-a), directed against the intracellular domain was used (**Figure 1A**). For comparison, the size of a primary-secondary-antibody-dye stack is depicted (**Figure 1B**). STED microscopy which shows a much more precise local resolution of each fluorophore was employed. As a result, compared to already high-resolution confocal images co-localization values for each protein were about 2-3 times lower (**Figure 1C**). In addition, software settings were adjusted to recognize co-localization when at least 50 pixels were overlapping to minimize background staining which reduced co-localization values by about 8-fold compared to a pixel by pixel matching.

### Distribution and Co-Localization of Desmosomal Proteins Vary Across the Different Epidermal Layers

**Figure 2:**
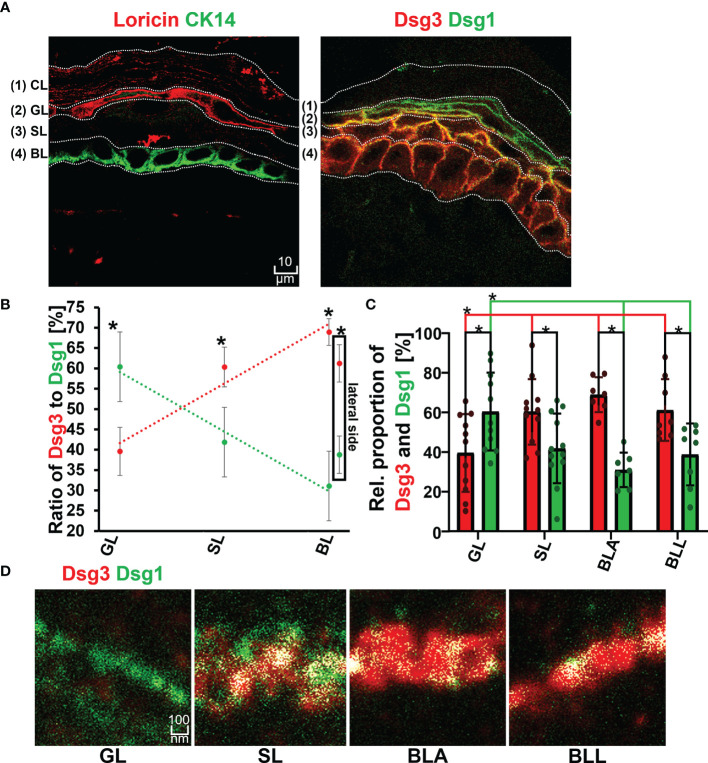
**(A)** Confocal microscopy images showing the identification of layers by staining of Loricin (red) and Cytokeratin 14 (Ck14 green) on the left and distribution of Dsg1 (green) and Dsg3 (red) along the epidermal layers and right. **(B)** Relative proportion of Dsg1 and Dsg3 along the different epidermal layers. **(C)** quantification of relative stained area of Dsg1 and Dsg3 along the epidermal layers. **(D)** STED microscopy images of the double staining borderof Dsg3 (red) and Dsg1 (green) at a single cell border, along the different epidermal layers. N (body donors)=3, n (cell borders)=2-7. *Significant difference to the value which is indicated that it is compared to.

The first set of experiments was carried out using the *ex vivo* human skin model as described before (21). Immunostaining for the two layer-specific markers loricrin and keratin14 were suitable to give a good indication for the granular layer (GL) and basal layer (BL) in human epidermis, respectively (**Figure 2A**). The staining of the two proteins in two consecutive sections from the same donor sample was used to differentiate between GL, spinous layer (SL) and BL. A gradient with increasing ratios of Dsg1 to Dsg3 staining towards more superficial layers was found (**Figures 2B** and ** S1**). This revealed the well-known superficial localization of Dsg1 and the more basal-dominant localization of Dsg3 observed in patient skin biopsies (1). Both Dsg 1 and Dsg3 showed a relatively uniform distribution along cell borders. No major differences were observed between the apical (BLA) and lateral side (BLL) of keratinocytes in the basal layer. Similarly, the desmosomal plaque proteins Dp and Pg showed a mostly uniform distribution across all epidermal layers.

**Figure 3:**
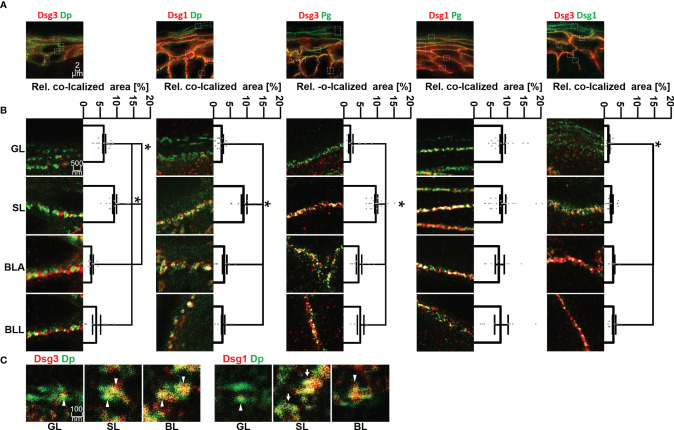
**(A)** Confocal microscopy images of co-stainings of Dsg3 or Dsg1 (red) with Dp, Pg or Dsg1 (green). **(B)** STED microscopy images of zoom to single cell border of the different epidermal layers. Shown to the right are quantifications of the co-localization of the two stained proteins. **(C)** STED microscopy images of co-stainings of Dsg3 or Dsg1 (red) with Dp (green) in human skin samples, sowing a resolution on a single desmosome scale. White pointers domains containing only either Dsg1 or Dsg3, white arrows whole desmosomes, containing almost only Dsg1 or Dsg3. N (body donors)=5, n (cell borders)=2-6. *Significant difference to the value which is indicated that it is compared to.

The co-localization of Dsg1 with Dsg3 did not show a correlation with the expression ratio of Dsg1 to Dsg3 and instead showed a linearly decreasing trend from basal to apical (**Figures 2D**, **3A, B** right most column). In the spinous layer (SL) the proportion of the two proteins was the most equal (**Figures 2B, C**). Despite the low amount of Dsg1 in the basal layer, the co-localization with Dsg3 was the highest for all layers. No difference between BLA and BLL was observed.

Co-localization of Dsg3 with Dp showed no significant difference between BLA and BLL but increased significantly towards SL and GL. However, co-localization of Dsg3 and Dp in the GL was still significantly higher than in the basal layer (**Figures 3A, B**, left column). For Dsg1, the situation was comparable with no significant difference in co-localization with Dp between BLA and BLL and the highest degree of co-localization within SL. However, co-localization of Dsg1 with Dp in the GL was similarly low as within the BL (**Figures 3A, B** second column).

In most layers, co-localization of Dsg1 and Dsg3 with Pg was higher than with other desmosomal components. Co-localization of Dsg3 with Pg was highest in SL and relatively lower in all other layers (**Figures 3A, B** third column). In contrast, for Dsg1 a relatively higher and constant co-localization with Pg across all epidermal layers was observed (**Figures 3A, B** fourth column).

With STED microscopy it was possible to achieve a resolution on a single desmosome level (**Figure 3C**). The desmosomes featured the typical structure with two neighbouring areas of Dp immunostaining labelling the desmosomal plaques. On this scale, desmosomes (**Figure 3C** white arrows) or domains which contain either one of the two isoforms only (**Figure 3C** white arrow heads). Desmosomes in GL displayed very little co-staining of Dsg1 or Dsg3 with Dp (**Figure 3**).

### In Pemphigus Patient Skin, Desmosome Number Was Altered More Than Desmosome Composition

**Figure 4:**
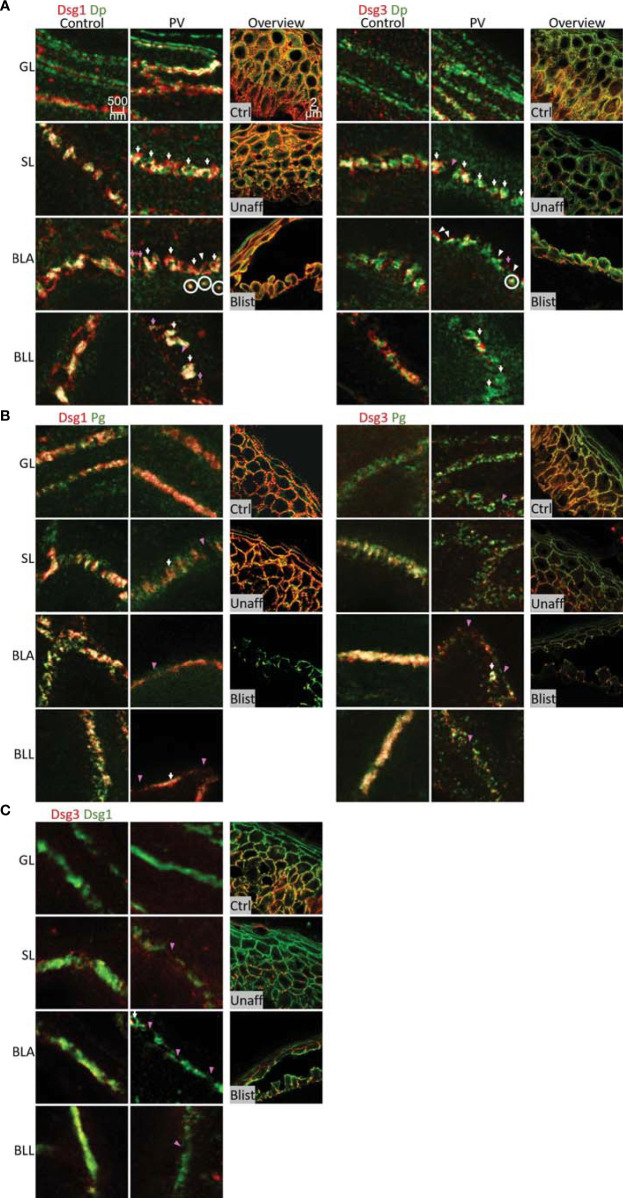
STED microscopy images of single cell borders from control vs. PV patient skin, and confocal image for overview of control, unaffected PV skin and blister region of PV skin. Co-stained for: **(A)** Dsg1 or Dsg3 (red) and Dp (green). **(B)** Dsg1 or Dsg3 (red) and Pg (green). **(C)** Dsg3 (red) and Dsg1 (Green). Fragmented and depleted Dsg3 staining pink arrows, clustered Dsgs white arrows, Fragmented desmosomespink pointers, split desmosomes white pinters, cytoplasmic Dsg-Dp-units white circles. N (body donors)=5, n (cell borders)=2-6.

**Figure 5:**
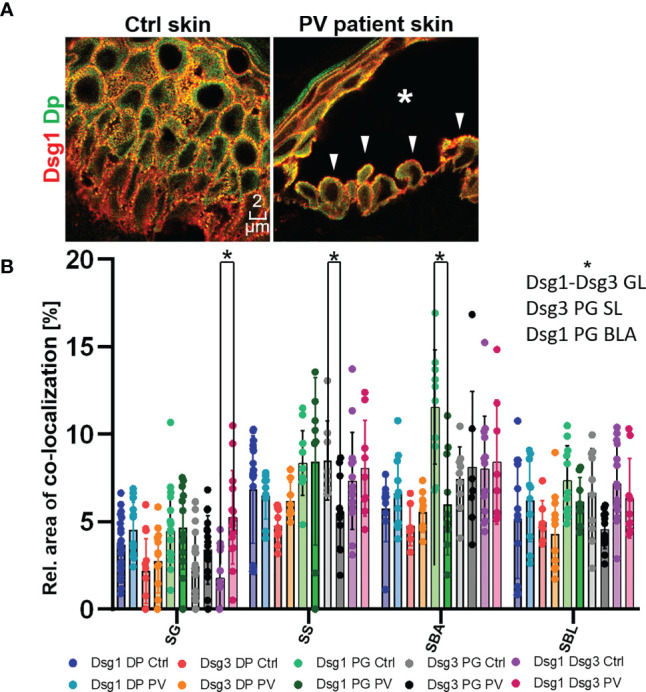
**(A)** Confocal microscopy images of a Dsg1 (red) Dp (green) co-staining in control and PV patient skin (featuring a typical PV blister * and tombstoning cells: white pointers). **(B)** quantification of differences in co-localization of desmosomal proteins in different layers in pemphigus patients compared to control patients N (patients)=3, n (cell borders)=2-7. *Significant difference to the value which is indicated that it is compared to.

In the second set of experiments, samples obtained from pemphigus vulgaris (PV) patients were studied. Lesions with typical pemphigus blister morphology were observed microscopically (**Figures 4**, **5A**). The blisters were located in the basal-suprabasal interface of the epidermis and showed typical tombstoning (**Figure 5A**, white arrow heads). Especially in blister regions, desmosomal structure and composition was altered. Dsg3 staining appeared fragmented and depleted from cell borders (**Figure 4** pink arrow heads), Dsg 1 and Dsg3 staining appeared clustered in some regions (**Figure 4** white arrows) and some desmosomes appeared to be fragmented (**Figure 4** pink arrows). In blister regions, split desmosomes were observed (**Figure 4** a left, white arrow heads). In some cells Dsg-Dp containing units, smaller than desmosomes, were observed localized to the cytoplasm away from cell borders (**Figure 4**, white circles). Second, co-localization of desmosomal components was investigated in pemphigus patient skin lesions (**Figure 5**). Significant changes were reduced co-localization of Dsg1 with Pg in BLA and of Dsg3 with Pg in SL, respectively. Furthermore, a higher co-localization of Dsg1 with Dsg3 in GL was observed for pemphigus patients. Despite clear morphological changes, there were no significant differences in the co-localization of other proteins in pemphigus patient compared to control skin, nor any significant changes in blister regions compared to non-blister regions.

**Figure 6:**
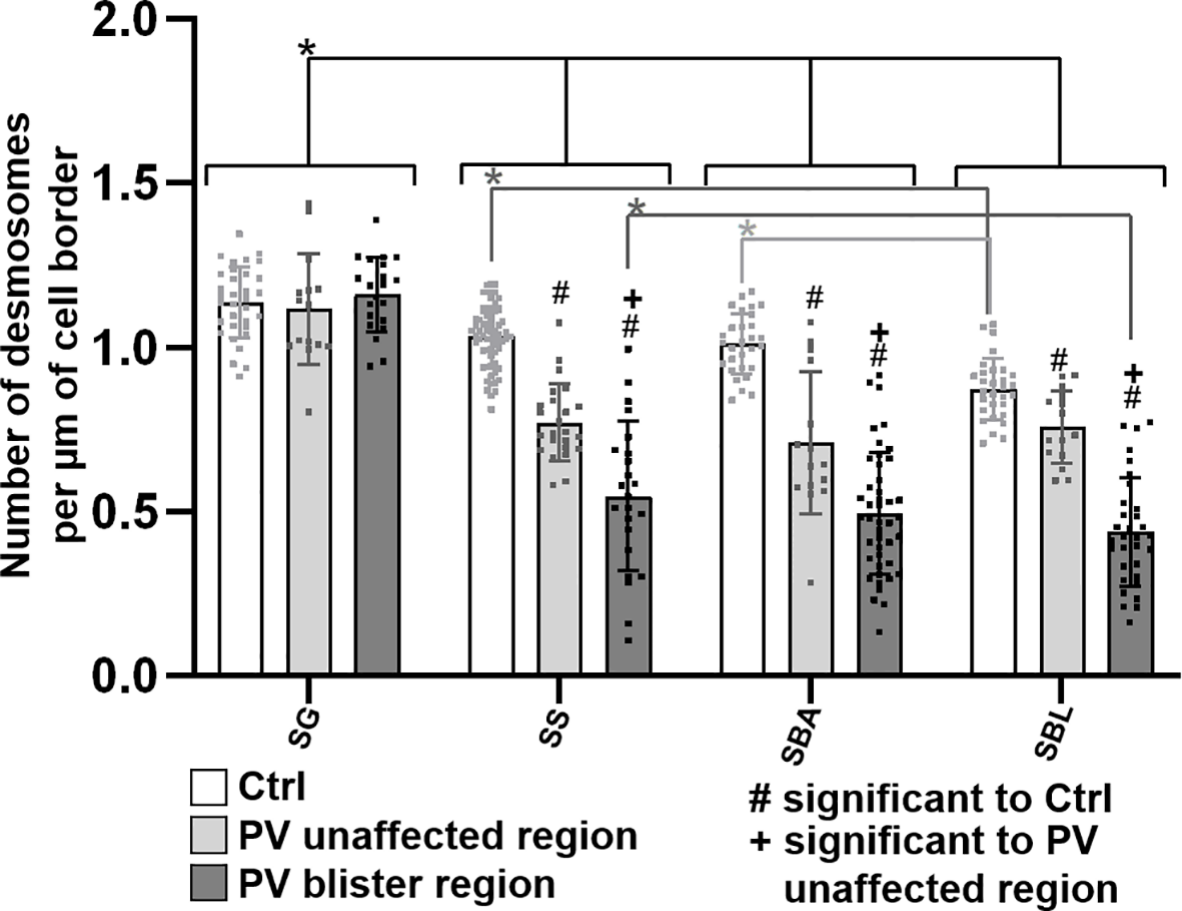
Quantification of desmosomes per membrane length in pemphigus patient and control skin. N (patients) = 3, n (cell borders) =1-7. *Significant difference to the value which is indicated that it is compared to.

Third, the number of desmosomes in patient skin compared to controls was quantified (**Figure 6**). In both control and PV skin, the density of desmosomes along cell borders across the epidermal layers constantly and significantly increased towards the more superficial layers. In controls in BLA, the number of desmosomes per µm of cell border was significantly higher than for BLL indicating that at the basal-suprabasal interface more desmosomes are present than along the lateral membranes of basal layer keratinocytes. In patient skin, the number of desmosomes in SL, BLA and BLL was significantly reduced especially in blister regions. Only exception was GL, where the number of desmosomes was not altered in PV skin (**Figure 6**).

## Discussion

**Figure 7:**
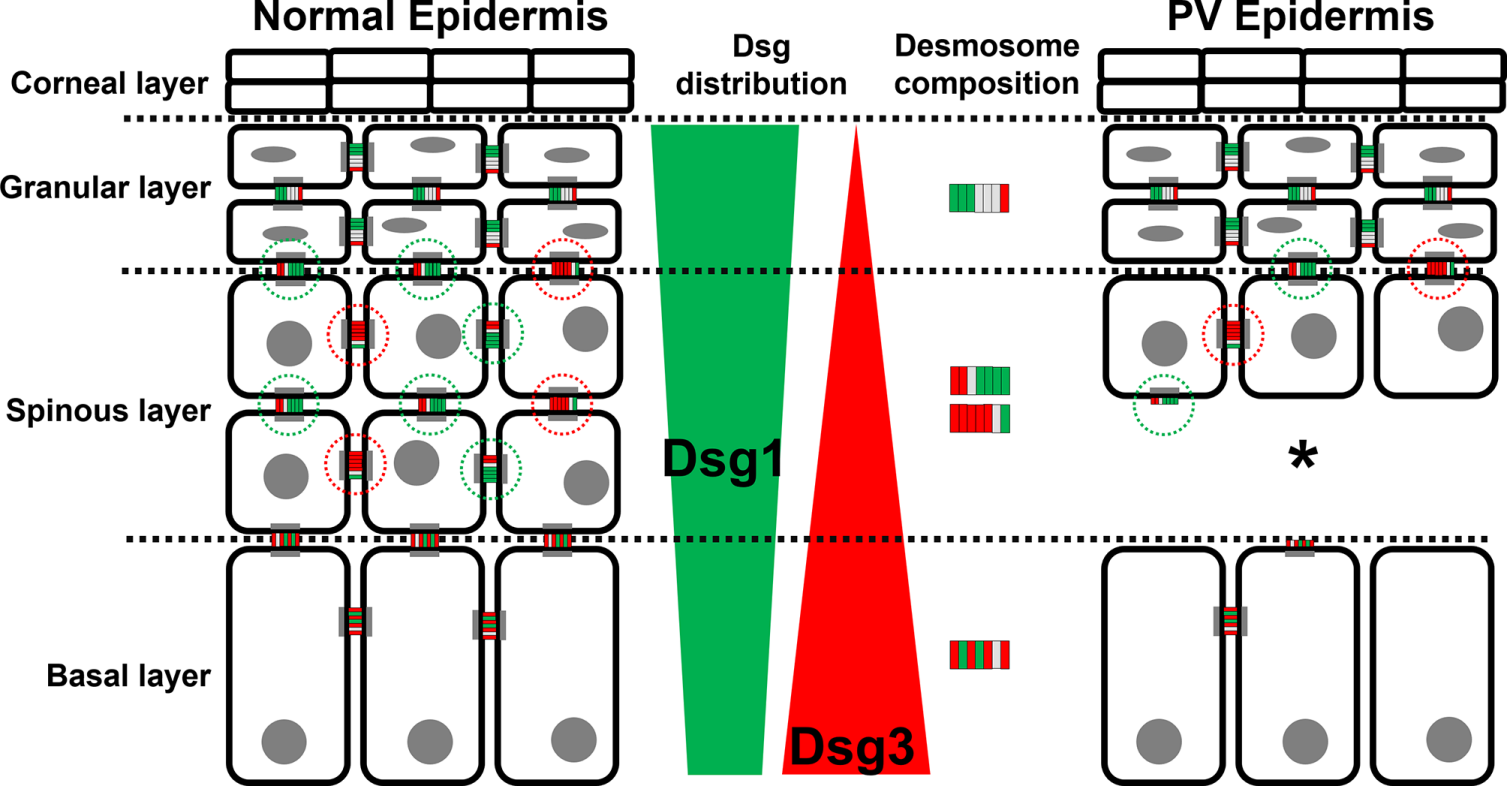
Schematic depiction of the distribution of the PV autoantigens Dsg1 (green) and Dsg3 (red) and the composition of desmosome along different epidermal layers in normal epidermis (left) and PV-affected epidermis (right). *Significant difference to the value which is indicated that it is compared to.

In this study we characterized the composition of desmosomes in the different epidermal layers in healthy skin and compared changes to PV patient samples. We focused on co-localization of the pemphigus autoantigens Dsg1 and Dsg3 with plaque proteins Pg and Dp. The main findings of this study are that the desmosomes in different layers of the epidermis vary regarding their composition (**Figure 7**). Moreover, we found that in lesional PV epidermis the number of desmosomes is reduced significantly in both basal and spinous layers where extradesmosomal Dsg1 and Dsg3 were depleted. These findings are in line with disturbed desmosome assembly during pemphigus pathogenesis and correlate with acantholysis along the basal-suprabasal interface in PV.

### Desmosome Composition Changes During Keratinocyte Differentiation

The current literature describes that Dsg3 is expressed more in the lower epidermis whereas Dsg1 expression increases during keratinocyte differentiation towards superficial epidermal layers (7, 22–24). The findings of this study confirm these expression patterns of Dsg1 and Dsg3. For Dsg1 a significant increase was observed towards GL only whereas SL and BL had a very similar Dsg1 content. In this context, it was shown that Dsg1 downregulates EGFR signalling and thereby reduces keratinocyte proliferation and allows keratinocyte differentiation (25, 26). In parallel, Dsg1 *via* ErbB2 facilitates tight junction assembly and barrier formation in the granular layer (27–29).

We observed that the composition of desmosomes in the different epidermal layers varies with respect to co-localization of Dsg1 and Dsg3 with each other and with the desmosomal plaque proteins Pg and Dp (**Figure 7**). Interestingly, co-localization of Dsg1 with Dsg3 in basal layer desmosomes was higher than in the superficial epidermis. Especially in the spinous layer, desmosomes which contained either Dsg1 or Dsg3 were observed. The co-localization of Dsg1 with Dsg3 was generally relatively low in all layers (**Figure 3A** right column), which can in part be explained by the used antibodies, since the targeted domains are further apart than for the other antibody combinations (**Figure 1A**). However considering the size of one or especially two primary-secondary-antibody-dye stack (**Figure 1B**) this cannot explain the low values in full. This indicates, that Dsg1 and Dsg3 show little overlap overall.

The desmosomes in GL often appeared to contain only little of both Dsg1 and Dsg3, which indicates that the desmosomes contain a larger amount of other desmosomal cadherins such as Dsc1-3 or Dsg4. However, it needs to be considered that this phenomenon at least in part may result from decreased accessibility of the epitopes for the antibodies used because of the beginning transformation into corneodesmosomes by incorporation of proteins including loricrin as well as crosslinking of the extracellular domains by corneodesmosin (30, 31). These data indicate that desmosomes during keratinocyte differentiation across the different epidermal layers mature from contacts containing both Dsg1 and Dsg3 to more differentiated desmosomes where not all desmosomal cadherins are present equally.

Dp and Pg staining on the other hand appeared to be mostly uniform across all layers. In the spinous layer, both Dsg1 and Dsg3 were found to be localized together with Dp to a larger extent and thus are more confined to desmosomes compared to other layers. In contrast, co-staining of Dsg1 and Dsg3 with Pg was higher compared to Dp demonstrating that extradesmosomal cadherin pools were also present. Co-localization was equal and high for Dsg1 with Pg across all layers indicating that extradesmosomal Dsg1 is also abundant in basal and granular layer desmosomes. However, extradesmosomal Dsg3 appeared to be confined to the lower epidermis but in general less prominent since co-localization of Dsg3 with Pg and Dp were comparable.

These findings may have implications for the signalling function of desmosomes. Extradesmosomal Dsg1 and Dsg3 not only serve as a pool for the formation of new desmosomes but also have signalling functions which are at least in part different to mature desmosomes (32). Some signalling molecules including PKC and p38MAPK were found to be sequestered and regulated by keratin filaments in desmosomes although most of p38MAPK is found outside the cytoskeleton-bound protein pool (33–35). In contrast, Rho-GTPases, Src and EGFR were found in the extradesmosomal pool of Dsg3 and proposed to be involved in desmosome assembly (36, 37). Because signalling regulating desmosome-turnover is important for pemphigus pathogenesis (38), these results on desmosome composition may also be relevant for the alterations of desmosomes observed in PV lesional skin.

### Desmosomes and Extradesmosomal Dsg Molecules Are Depleted Predominantly in the Lower Epidermis in PV Indicating Disturbed Desmosome Assembly

The histology observed for the pemphigus patient samples investigated in this study showed microscopically visible suprabasal blisters indicating a typical PV phenotype (1, 14). PV usually is associated with anti-Dsg3 and anti-Dsg1 autoantibodies which cause suprabasal blistering whereas autoantibodies against Dsg1 are believed to cause superficial blistering of the epidermis as observed in PF (10, 15). In the granular layer, we confirmed that Dsg3 expression was low and desmosomes contained Dsg1 presumably together with other desmosomal cadherins. This result would fit well to the concept that autoantibodies targeting Dsg1 alone in PF would cause superficial splitting as proposed by the desmoglein compensation hypothesis (39).

We found that in PV skin desmosomes were significantly predominantly reduced in the basal and spinous layer keratinocytes which is in line with previous findings (40). This holds true for sites of blister formation but also unaffected skin indicating that loss of desmosomes may indeed be a predominant factor for blister formation as suggested from human skin *ex vivo* models (21, 41). Pemphigus is a disease caused by disturbed turnover of desmosomes in which both assembly and disassembly were proposed to be affected (42, 43). In line with this, we found that extradesmosomal Dsg1 was significantly reduced at the basal-suprabasal interface and extradesmosomal Dsg3 was reduced in spinous layer keratinocytes indicating that loss of desmosomes in the lower epidermis may at least in part be caused by impaired desmosome assembly. In addition, split desmosomes were also found similar to *ex vivo* models (41) and other studies in patient skin (40, 44). The fact that basal desmosomes appeared to be immature and to contain both Dsg1 and Dsg3 may help to explain why autoantibodies against both isoforms are usually required for epidermal blistering in PV. On the other hand, no differences were found in the composition of desmosomes from the apical and the lateral side of basal layer keratinocytes both in control skin as well as in pemphigus patients. In contrast, in the *ex vivo* pemphigus model, apical but not lateral desmosomes were found to be preserved significantly when Erk was inhibited in parallel to incubation with PV-IgG (41).

Also, the loss of extradesmosomal Dsg1 and Dsg3 in the lower epidermis in PV lesions may reflect the role of signalling in epidermal blister formation. After incubation with PV-IgG and PF-IgG, both p38MAPK and Erk were found to be activated in the extradesmosomal pool whereas Src activation was confined to the desmosomal pool (45). The data presented here demonstrate that extradesmosomal Dsg1 and Dsg3 are prevalent at the basal-suprabasal interface where acantholysis is found in PV whereas in the granular layer, the site of epidermal splitting in PF, extradesmosomal Dsg1 exceeds the amount of Dsg1 localized to desmosomes. The loss of extradesmosomal Dsg1 and Dsg3 in PV skin correlates well with these signalling functions. Because antibodies against Dsg1 are known to be important for epidermal blistering and Dsg1 was reported to regulate signalling mechanisms in part differently compared to Dsg3 such as activation of the PLC/Ca^2+^ pathway, the prevalence of extradesmosmal Dsg1 at sites of blister formation in PV and PF would explain differences in the clinical phenotypes of pemphigus.

## Materials and Methods

### Human Skin Samples

Skin biopsies ~4 cm² was excised from the shoulder region of body donors deceased for less than 24 h and divided into ~0.25 cm² pieces and frozen in tissue freezing medium (Leica, Wetzlar Ger). Patient skin samples were obtained as frozen Blocks from the Charité Berlin.

Skin samples were cut into 6 µm thick slices and transferred to silicate glass coverslips for further processing.

### Immunostaining

Samples were heated to 60°C for 30 min, washed with PBS and fixed with 8% glyoxal (with 20% ethanol in water at a pH of 4-5) for 30 min at room temperature (RT) or in ethanol (-20°C) shaking on ice for 30 min and acetone (-20°C) for 3 min. Glyoxal-fixed cells were permeabilized with 1% Triton X-100 in PBS for 45 min and blocked with 3% bovine serum albumin (BSA) and 1% normal goat serum in PBS for 30 min. Primary antibodies were applied over 3 h at RT STAR-RED- or Alexa 594-coupled goat-anti-rabbit/mouse secondary antibodies (Abberior GmbH, Göttingen, Ger) were incubated for 1 h and DAPI 1:10.000 for 15 min. The coverslips were mounted on glass slides using Prolong™ Diamond Antifade Mountant (Thermo Fisher GmbH Dreieich, Ger).

### STED Microscope

After immunostaining, cells were mounted using ProLong™ Diamond anti fade mountant (Thermo Fisher GmbH Dreieich, Ger) and imaged using an Abberior 3D Stimulated emission depletion (STED) confocal microscope with IMMOIL-F30CC (Olympu GmbH, München, Ger) Star Red and Alexa 594 were excited at 638  nm and 594 nm respectively using pulsed diode lasers (PDL 594, Abberior Instruments; PiL063X, Advanced Laser Diode Systems). Fluorescent molecules were depleted at 775 nm with a pulsed fibre laser (PFL-P-30-775B1R, MPB Communications) and emission was detected with an avalanche photodiode detector at 605-625 and 650-720 nm range.

### Statistical Analysis

Data were analysed using one/two-way-ANOVA using Graphpad Prism (Graphpad Software, USA). Brown-Forsythe test (one-way) and Barletts test (one-way) and correction and Tukeay correction for multiple comparisons was performed. Error bars represent SEM. Significance was assumed with p ≤ 0.05. Data are shown as mean ± SEM. Each n represents one independent experiment.

## Data Availability Statement

The original contributions presented in the study are included in the article/**Supplementary Material**. Further inquiries can be directed to the corresponding author.

## Ethics Statement

The studies involving human participants were reviewed and approved by Institute of Anatomy and Cell Biology, Ludwig-Maximilian-Universität München, Germany and Charité Berlin (Eithics vote Nr.: EA4/194/19). The patients/participants provided their written informed consent to participate in this study.

## Author Contributions

TS: concept, special methodology, general methodology, experiments, manuscript writing JP: experiments, special methodolgy LS: preparation of samples KM: providing samples KG: providing samples FV: providing samples DK: general methodology JW: concept, manuscript writing. All authors contributed to the article and approved the submitted version.

## Funding

The study was supported by DG (FOR 2497) to JW, FV and KG.

## Conflict of Interest

The authors declare that the research was conducted in the absence of any commercial or financial relationships that could be construed as a potential conflict of interest.

## Publisher’s Note

All claims expressed in this article are solely those of the authors and do not necessarily represent those of their affiliated organizations, or those of the publisher, the editors and the reviewers. Any product that may be evaluated in this article, or claim that may be made by its manufacturer, is not guaranteed or endorsed by the publisher.
