# Supplementary material for: Dsg1 and Dsg3 Composition of Desmosomes Across Human Epidermis and Alterations in Pemphigus Vulgaris Patient Skin
Source: Front Immunol. 2022 May 25;13:884241. doi: 10.3389/fimmu.2022.884241 (PMC9196036; doi:10.3389/fimmu.2022.884241)
Supplement: Supplementary file 1 [file DataSheet_1.docx]

**Antibody specifications**

hDsg1 NP_001933.2

mdwsffrvvamlfiflvvvevnsefriqvrdyntkngtikwhsirrqkrewikfaaacregednskrnpiakihsdcaanqqvtyrisgvgidqppygifvinqktgeinitsivdrevtpffiiycralnsmgqdlerplelrvrvldindnppvfsmatfagqieensnantlvmilnatdadepnnlnskiafkiirqepsdspmfiinrntgeirtmnnfldreqygqyalavrgsdrdggadgmsaececnikildvndnipymeqssytieiqentlnsnlleirvidldeefsanwmaviffisgnegnwfeiemnertnvgilkvvkpldyeamqslqlsigvrnkaefhhsimsqyklkasaisvtvlnviegpvfrpgsktyvvtgnmgsndkvgdfvatdldtgrpsttvryvmgnnpadllavdsrtgkltlknkvtkeqynmlggkyqgtilsiddnlqrtctgtininiqsfgnddrtntepntkittntgrqestsstnydtsttstdssqvyssepgngakdllsdnvhfgpagigllimgflvlglvpflmiccdcggaprsaagfepvpecsdgaihswavegpqpeprdittvipqippdnaniiecidnsgvytneyggremqdlgggermtgfeltegvktsgmpeicqeysgtlrrnsmrecregglnmnfmesyfcqkayayadedegrpsndclliydiegvgspagsvgccsfigedlddsfldtlgpkfkkladislgkesypdldpswppqstepvclpqetepvvsghppisphfgtttvisestypsgpgvlhpkpildplgygnvtvtesyttsdtlkpsvhvhdnrpasnvvvtervvgpisgadlhgmlempdlrdgsnvivterviapssslptsltihhpressnvvvterviqptsgmigslsmhpelanahnvivtervvsgagvtgisgttgisggigssglvgtsmgagsgalsgagisgggiglsslggtasighmrsssdhhfnqtigsaspstarsritkystvqysk

Extracellular

Transmenbrane

Intracellular

Anti-Dsg1 Abclonal (A9812) aa800-1049

hDsg3 NP_001935.2

mmglfprttgalaifvvvilvhgelrietkgqydeeemtmqqakrrqkrewvkfakpcregednskrnpiakitsdyqatqkityrisgvgidqppfgifvvdkntgdinitaivdreetpsflitcralnaqgldvekpliltvkildindnppvfsqqifmgeieensasnslvmilnatdadepnhlnskiafkivsqepagtpmfllsrntgevrtltnsldreqassyrlvvsgadkdgeglstqcecnikvkdvndnfpmfrdsqysarieenilssellrfqvtdldeeytdnwlavyfftsgnegnwfeiqtdprtnegilkvvkaldyeqlqsvklsiavknkaefhqsvisryrvqstpvtiqvinvregiafrpasktftvqkgisskklvdyilgtyqaidedtnkaasnvkyvmgrndggylmidsktaeikfvknmnrdstfivnktitaevlaideytgktstgtvyvrvpdfndncptavlekdavcssspsvvvsartlnnrytgpytfaledqpvklpavwsittlnatsallraqeqippgvyhislvltdsqnnrcemprsltlevcqcdnrgicgtsypttspgtrygrphsgrlgpaaigllllgllllllaplllltcdcgagstggvtggfipvpdgsegtihqwgiegahpedkeitnicvppvtangadfmessevctntyargtavegtsgmemttklgaatesggaagfatgtvsgaasgfgaatgvgicssgqsgtmrtrhstggtnkdyadgaismnfldsyfsqkafacaeeddgqeandclliydnegadatgspvgsvgccsfiaddlddsfldslgpkfkklaeislgvdgegkevqppskdsgygiescghpievqqtgfvkcqtlsgsqgasalstsgsvqpavsipdplqhgnylvtetysasgslvqpstagfdplltqnvivtervicpissvpgnlagptqlrgshtmlctedpcsrli

Extracellular

Transmenbrane

Intracellular

Anti-Dsg3 (a) Biozol (ELA-E-AB-62720-120) aa749-999

Anti-Dsg3 (b) Invitrogen (32-6300)

hPg AAG16727.1

mevmnlmeqpikvtewqqtytydsgihsgantcvpsvsskgimeedeacgrqytlkktttytqgvppsqgdleyqmsttarakrvreamcpgvsgedsslllatqvegqatnlqrlaepsqllksaivhlinyqddaelatralpeltkllndedpvvvtkaamivnqlskkeasrralmgspqlvaavvrtmqntsdldtarcttsilhnlshhregllaifksggipalvrmlsspvesvlfyaittlhnlllyqegakmavrladglqkmvpllnknnpkflaittdclqllaygnqeskliilanggpqalvqimrnysyekllwttsrvlkvlsvcpsnkpaiveaggmqalgkhltsnsprlvqnclwtlrnlsdvatkqeglesvlkilvnqlsvddvnvltcatgtlsnltcnnsknktlvtqnsgvealihailragdkdditepavcalrhltsrhpeaemaqnsvrlnygipaivkllnqpnqwplvkatiglirnlalcpanhaplqeaaviprlvqllvkahqdaqrhvaagtqqpytdgvrmeeivegctgalhilardpmnrmeifrlntiplfvqllyssveniqrvaagvlcelaqdkeaadaidaegasaplmellhsrnegtatyaaavlfrisedknpdyrkrvsveltnslfkhdpaaweaaqsmipinepygddmdatyrpmyssdvpldplemhmdmdgdypidtysdglrppyptadhmla

Anti-Pg Progen (61005)

Anti-Dp Progen (61024)


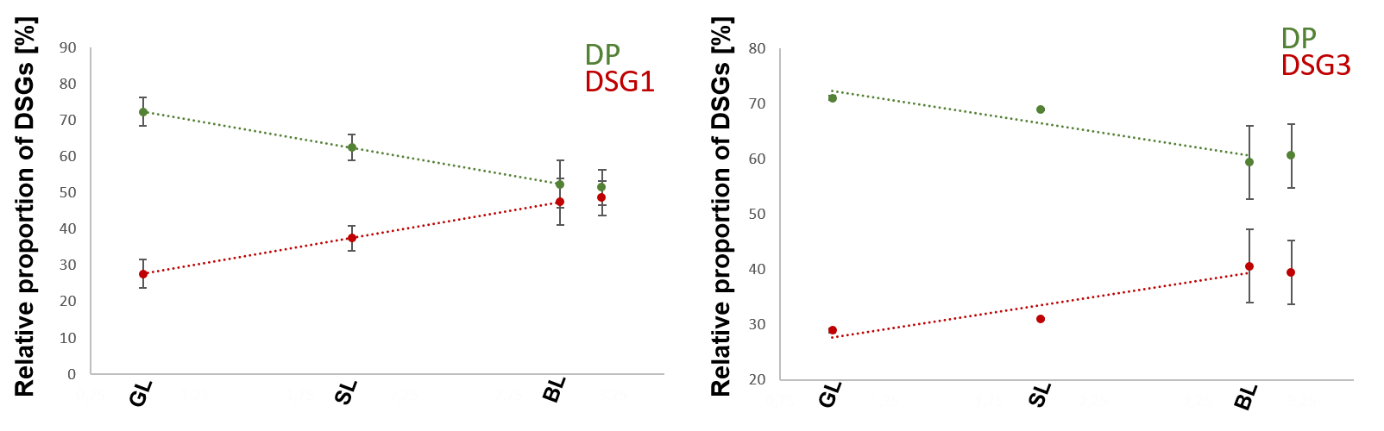


**Fig S1**: Ratio between Dsg1/Dsg3 staining (red) and Dp staining (green) in different layers of the epidermis, determined using STED microscopy on single cell borders. N (bodydonors) = 5, n (cell borders) = 2-6.


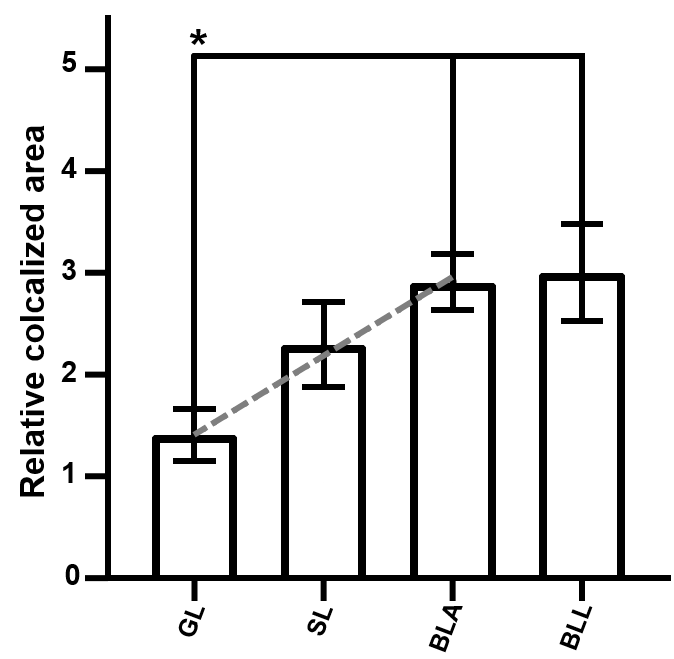


**Fig S2**: Quantification of co-localization of Dsg1 with Dsg3 along the different epidermal layers showing a strictly decreasing linear trend. N (bodydonors) = 5, n (cell borders) = 2-6.

**Table S1:** Serological data for Pemphigus vulgaris (PV) patient samples.

| **Patient** | **Date of sampling** | **Dsg1 Score*** | **Dsg3 Score*** | **Diagnosis** |
| --- | --- | --- | --- | --- |
| 1 | Feb. 05.2021 | <2 | 89 | PV |
| 2 | Feb. 18.2021 | 126 | >200 | PV |
| 3 | Mar. 17.2021 | n.a. | n.a. | PV |

*Serology score (titer) determined by enzyme immunoassay
